# ProtGraph: a tool for the quick and comprehensive exploration and exploitation of the peptide search space derived from protein sequence databases using graphs

**DOI:** 10.1093/bib/bbae671

**Published:** 2025-01-05

**Authors:** Dominik Lux, Katrin Marcus-Alic, Martin Eisenacher, Julian Uszkoreit

**Affiliations:** Ruhr University Bochum, Medical Faculty, Medizinisches Proteom-Center, Gesundheitscampus 4, 44801 Bochum, Germany; Ruhr University Bochum, Medical Faculty, Center for Protein Diagnostics (PRODI), Gesundheitscampus 4, 44801 Bochum, Germany; Ruhr University Bochum, Medical Faculty, Medizinisches Proteom-Center, Gesundheitscampus 4, 44801 Bochum, Germany; Ruhr University Bochum, Medical Faculty, Center for Protein Diagnostics (PRODI), Gesundheitscampus 4, 44801 Bochum, Germany; Ruhr University Bochum, Medical Faculty, Medizinisches Proteom-Center, Gesundheitscampus 4, 44801 Bochum, Germany; Ruhr University Bochum, Medical Faculty, Center for Protein Diagnostics (PRODI), Gesundheitscampus 4, 44801 Bochum, Germany; Ruhr University Bochum, Medical Faculty, Core Unit Bioinformatics - CUBiMed.RUB, Universitätsstr. 105, 44789 Bochum, Germany; Ruhr University Bochum, Medical Faculty, Core Unit Bioinformatics - CUBiMed.RUB, Universitätsstr. 105, 44789 Bochum, Germany; Ruhr University Bochum, Medical Faculty, Medical Bioinformatics, Universitätsstr. 105, 44789 Bochum, Germany

**Keywords:** variants, graphs, proteomics, bioinformatics

## Abstract

Due to computational resource limitations, in mass spectrometry based proteomics only a limited set of peptide sequences is used for the matching against measured spectra. We present an approach to represent proteins by graphs and allow not only the canonical sequences but also known isoforms and annotated amino acid variations, e.g. originating from genomic mutations, and further common protein sequence features contained in Uniprot KB or other protein databases. Our C++ and Python implementation enables a groundbreaking comprehensive characterization of the peptide search space, encompassing for the first time all available annotations in a protein database (in combination more than $10^{200}$ possibilities). Additionally, it can be used to quickly extract the relevant subset of the search space for peptide to spectrum matching, e.g. filtering by the peptide mass. We demonstrate the advantages and innovative findings of our implementation compared to previous workflows by re-analysing publicly available datasets.

## Graphical Abstract

**Graphical Abstract:**
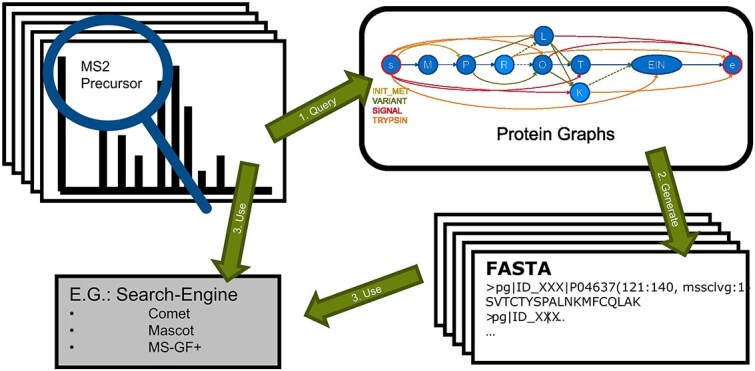

## Introduction

For the high throughput proteomics analysis of complex biological samples, liquid chromatography coupled to tandem mass spectrometry (LC-MS/MS) has become the state-of-the-art method [1, 2]. Especially bottom-up or shotgun proteomics is widely used, where the original proteins in a sample are enzymatically digested into peptides before measurement [3]. For the actual identification of mass spectra originating from measured peptides, so-called peptide search engines [4–7] are applied, which make use of protein sequence databases. Also for spectral library matches, another approach for identifying spectra, it is mandatory to first have either good peptide spectrum matches (PSMs) or at least predicted spectra for peptides of a specific mass range [8–11]. For all mentioned methods, the search space for spectra contains only sequences occurring in the original protein or peptide sequence databases, with the addition of a small number of allowed modifications or potential amino acid exchanges.

**Figure 1:**
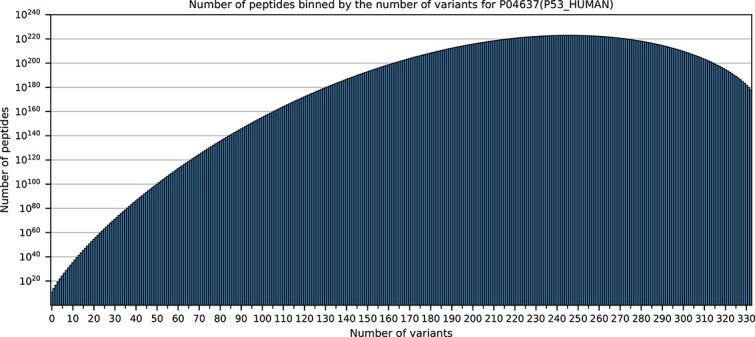
Total distribution of peptides binned by the number of variants per peptide for P04637 (P53_HUMAN). The plot was generated based on a protein graph containing variants/conflicts/mutagens and other processing information and was *in silico* digested by the enzyme Trypsin. It can be seen that the number of peptides without any variants per peptide is the lowest, while the total number of peptides created when taking exactly 246 variants per peptide into account is highest. The shown numbers account for up to infinite missed cleavages per peptide and amino acid sequence lengths from one to unlimited. The illustration shows that by restricting the number of variants per peptide e.g. to 3, the possible peptide search space is significantly reduced.

Most current workflows work with protein sequence databases, which are provided as FASTA-databases, a plain text export of amino acid sequences. These exports usually contain only a representative or “canonical” sequence of a protein, sometimes also annotated isoforms, if provided by the database. Many resources, like the UniProtKB [12], contain additionally to the canonical sequences annotations of single amino acid polymorphisms (SAPs), which usually derive from single nucleotide polymorphisms (SNPs) in the genome. Furthermore, processing information like the cleavage of initial methionine, signal- or pro-peptides or other effects due to the maturation of proteins may be annotated. Still, the edited or matured sequences are commonly not exported to the search algorithms and thus are not accounted for during spectrum identification. One of the reasons why such an export is not trivial is the combinatorial problem: if for a protein or peptide two SAPs on different locations are annotated, four sequences need to be considered for the export. For well annotated proteins, this combinatorial explosion already reaches millions of possible generated peptides per protein. We exemplary listed the number of all possible peptides per allowed number of variants for the very well studied and annotated human P53 in Fig. 1. Thus, a direct export of all possible SAP combinations becomes unfeasible, even though the PEFF format [13] allows the export of these annotations and first search engines are starting to adopt PEFF as database input, but limit the number of incorporated annotations. For specific species, special resources were developed in the past which contain only sequences accounting for the incorporation of single variants or specific variants, which were analysed by different approaches like transcriptomics [14], though a complete integration of all possible combinations of annotated variants and protein processing information was not published before.

A graph representation of proteins is a suitable and accessible solution to this problem, as was shown before for variations in genomics [15, 16]. We demonstrate an implementation that models any annotated protein sequence information available in UniProtKB, like SAPs, exchanges of longer sequences, and any maturation processes. The proposed implementation can furthermore model cleavage sites for multiple enzymes including unspecific cleavages (i.e. creation of any possible peptide subsequence), can quickly retrieve information of the total number of possible peptides and allows very quick searching of peptides in a graph on certain criteria like the amino acid sequence or mass. Furthermore, this graph-representation can be exploited for the export of filtered peptides to allow the spectrum wise identification of current search engines used in mass spectrometry based proteomics.

## Materials and methods

### Graph definition and overview of the graph generation

**Figure 2:**
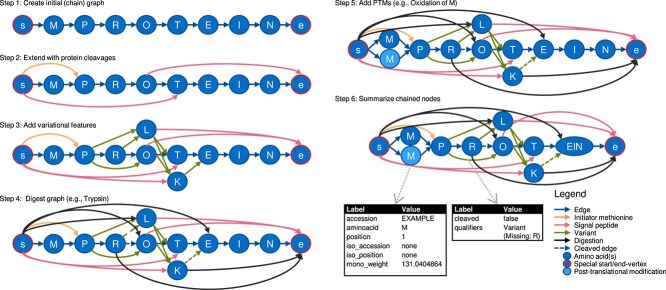
Brief overview of the graph generation. First, an initial protein-graph is generated, with its corresponding start and end vertex (step 1). It is further appended with additional Information, like feature-information from UniProtKB provided by the UniProt flat file format as well as digestion and post-translational-modification information. Step 6 showcases the optimization step concatenating three vertices (and two edges) into a single vertex. All available labels of the protein-graph are shown on the bottom right.

Throughout this work, we introduce and use protein-graphs. These graphs describe canonical and isoform protein sequences with additional annotated features. In essence, a protein-graph is a labelled, directed, and acyclic graph $G = (V, E)$, where $V \subset \mathbb{N}_{0}$ is the set of vertices and $E \subseteq (V \times V)$ the set of edges. The vertices and edges are enumerated with unique identifiers, which are used to access the annotated labels of the corresponding vertices and edges. One important label of the vertices is the actually contained amino acids. A full list of all labels for vertices and edges is provided on the bottom right in Fig. 2.

As a source to generate protein-graphs, formatted text files from the UniProtKB are used (downloadable *.txt-files or “UniProt flat files”). These files contain canonical as well as isoform sequences of proteins and include furthermore additional features like variations on the sequence, information on cleaved peptides during protein maturation, structural information (a-helix, b-sheets), and/or information about common post-translational modifications like phosphorylation. While parsing these entries we initially extract the canonical as well as any isoform sequences. These sequences are then used to generate the initial protein-graph $PG_{i}$ (illustrated in step 1 in Fig. 2). Here, for each amino acid in the sequence a vertex is created, consecutive edges are added in between the created vertices, connecting them which we will refer to as a chain of vertices. For each isoform an additional chain of vertices is created. Additionally, a dedicated start and end vertex ($s$ and $e$, respectively) is added to the graph, the beginning of the chain(s) are connected to $s$, the ends to $e,$ respectively.

Due to dedicated start and end vertices, valid sequence-paths MUST start from $s$ and end in $e$ to retrieve valid protein sequences or, after information about (enzymatic) digestion is added, peptide sequences. A sequence then can be retrieved by concatenating the amino acid labels along a complete sequence-path and would e.g. yield in $PG_{i}$ the canonical or an isoform protein-sequence, depending on which path was taken. All other paths, which do not start in $s$ or end in $e$, are considered as incomplete sequence paths in a protein-graph, since these paths describe only a section of the complete sequence. In the scope of this article we use the term peptide mainly as a synonym for “peptide sequence”, not actually meaning the biomolecule itself. More precisely, a peptide in this manuscript is given by its path through the protein-graph from its start and end vertex, after adding (enzymatic) digestion. This means, a peptide is not only defined by its amino acid sequence, but also by its position in the protein and any annotated information.

The initial protein-graph $PG_{i}$ is further extended using specific rulesets to encode annotated feature information. An overview of the applied order is illustrated in Fig. 2, detailed information for each step is given in the supplementary data in sections 1.1–1.3. The resulting protein-graph thus holds a concise and comprehensive representation of all possible proteins, respectively peptides, which can be generated by the given features and selected post-translational modifications.

More information on the optimization of the protein graphs and the actual algorithms to retrieve the peptide and protein information can be found in the supplementary data in section 1.4, following is a brief summation. We further optimize the graphs by drastically reducing their number of vertices and edges (Supplemental Listing 1) by identifying chains of nodes and summarizing these chains into single vertices. With these graphs, various characteristics (Supplemental Listing 2) can be retrieved, such as the number of peptides, by a specific property like length or specific feature (as shown in Fig. 1 by the number of variants). Finally, we implemented an algorithm (Supplemental Listing 3), which allows to efficiently query peptides in protein-graphs by providing a mass range in Dalton. The returned peptides entries from a set of protein graphs (e.g. species) can be then used to construct a precursor specific FASTA database, which is tailored to the dataset.

### Benchmarking datasets and workflow for spectrum matching

The protein graph generation is implemented in Python and made available on PyPi under the package name protgraph (https://github.com/mpc-bioinformatics/Protgraph, version 0.3.10). The Python packages Biopython (version 1.81) [17] and igraph 0.10.4 [18] are used to parse UniProt flat file formatted entries and to generate protein graphs, respectively. Multiple other Python packages are integrated into ProtGraph to export protein graphs in various formats. The algorithm in Supplemental Listing 3 has been implemented in C++ 20 to efficiently generate precursor specific FASTA entries from protein graphs.

To exploit the protein-graphs and their stored annotations, we developed two workflows using ProtGraph to export peptides into FASTA format for subsequent spectrum identification with common peptide spectrum matching algorithms, like Comet [6] or MS-GF+ [7]. All workflows used in this work (including the FASTA generation from protein-graphs and identification) are implemented in Nextflow (version 23.4.2) [19] using the DSL 2 syntax and are combined to provide three different identification workflows: a protein-FASTA (containing only canonical protein sequences), a global FASTA (without features for comparison), and a precursor specific FASTA (with all features, except MUTAGEN) is used (https://github.com/mpc-bioinformatics/ProGFASTAGen/) [20]. The workflows utilize the ThermoRawFileParser v1.4.2 [21] for conversion and Comet v2023.01.2 [6] with Percolator 3.06 [22] for identification. The protein graphs, which are generated for the precursor specific FASTA, are exported in a binary compressed sparse row format (CSR, where the nodes are already sorted in topological order), which is then used in combination with a query csv file by the C++ implementation in order to retrieve the FASTA.

The first workflow builds protein-graphs containing all possible peptides from a given database, limited by global attributes like peptide length or allowed miscleavage sites (global workflow). This workflow (shown in Supplemental Fig. 2a)) executes ProtGraph, generates characteristics regarding the number of exported peptides, and exports these peptides into a FASTA file, containing selected feature combinations. As some protein-graphs contain unfeasible amounts of peptide sequences, like shown in the prior paragraphs, it is not possible to export all possible peptides, while considering variant-features. For this we constructed a second workflow which uses ProtGraph, the precursor m/z-values of MS2-spectra, and a C++-Implementation of a more sophisticated graph-traversal algorithm to generate spectrum precursor mass specific FASTA databases. This algorithm is based on a target-value search [23], which was extended to retrieve peptides fitting to an interval-query, generated from the precursors’ spectra (see Supplemental Listing 3 for a complete explanation of the algorithm). The calculation of the algorithms complexity is given in section 4 in the supplementary document and results in the worst case in $\mathcal{O}(2^{n-1})$. In this precursor-specific workflow (Supplemental Fig. 2b), protein-graphs are created as in the first workflow, followed by a traversal of the protein-graphs saving only peptides, which match the masses of the precursors for MS2-spectra under consideration. For some very well-annotated proteins, even this approach leads to infeasible amounts of peptides for an export. Therefore, the possible number of features per peptide in a given mass bin, which still allows a feasible export, can be estimated and applied before the export (see Supplemental Fig. 1).

We benchmarked both workflows and compared them to a common identification workflow, which uses an unmodified human proteome FASTA downloaded from UniProt as reference (19.06.2023, Proteome-ID UP000005640, UniProt Release 2023_02). For the analyses, two datasets were downloaded from PRIDE [24]: PXD002171, in which human hepatocellular carcinoma samples were analysed on an LTQ Orbitrap Elite (HCC dataset), and PXD028605, where human whole blood samples were analysed on a Q Exactive HF (blood dataset).

The workflows have been set up on a single server (2TB RAM, 2x AMD EPYC 7452 32-Core) and were applied to the datasets using the human proteome-database (downloaded at 19.06.2023, Proteome-ID UP000005640, UniProt Release 2023_02) as FASTA and UniProt flat files for the corresponding workflows, containing 82 492 proteins. For the HCC dataset, we allowed for the precursor specific FASTA generation and identification of the oxidation of M and propionamide on C as variable modifications, and set a precursor mass tolerance of 5 ppm. For the blood dataset, fixed carbamidomethylation of C and variable oxidation of M was set and a precursor mass tolerance of 10 ppm was used. Further, we limited the precursor specific and global FASTA for both datasets to contain only peptides with a mass of up to 5000 Da. We used Comets internal decoy-generator, which generates for each peptide in the database the reversed sequence and used the q-value based on the sorted entries provided by Percolator per measurement and used a cutoff value of 1% for the results.

## Results

### Generating protein graphs with ProtGraph

We developed ProtGraph, which is able to store every protein entry in the UniProtKB into a graph structure as protein-graphs. These directed and acyclic graphs contain in a compact representation the information about the canonical and isoform-sequence of proteins as well as annotated features like amino acid variations, conflicts (a special kind of variation), mutagenic variations, cleavage sites such as signal-, peptide-, and pro-peptide-information due to protein maturation and localization. ProtGraph creates these graphs in a highly parallel manner and is therefore able to generate protein-graphs for arbitrary-sized species-databases in a short time. Running ProtGraph on the whole UniProtKB (version 2023_04), which contains 244.4 million proteins, and mapping all parsable features into protein-graphs needs less than 1 day on a single server (see 5 Methods section for server specifications). ProtGraph performs the steps explained in the 5 Methods section to further reduce the graph size by summarizing chains of vertices (i.e. common amino acid sequences) into single vertices. This optimization reduces the total number of vertices from 86 to 9.5 billion (by 89%) and the number of edges from 103 to 26 billion (by 74%) for the entire UniProtKB. This illustrates that the resulting protein-graphs mostly consist of chains of vertices, while still maintaining all annotated feature-information.

### Characterization of protein-graphs

Especially for well-annotated species like human and mouse, an export into the FASTA format for the identification of all possible combinations of variants and features can still be unfeasible. To quickly explore the actual number of possible peptides, we implemented a dynamic programming approach on protein-graphs. With this approach, information about the total number of peptides as well as the total number of peptides grouped by an attribute, like peptide-length or the numbers of missed enzymatic cleavage sites, variants, mutagens, conflicts, and other features, can be retrieved per protein-graph.

First we investigated the protein-graphs with the variant-features and found that the total number of possible peptides is dominated by a single protein: the well studied human tumour protein P53 (P04637). This protein is the most annotated protein in UniProtKB, containing 1363 annotated variants, while having a canonical sequence length of only 393 amino acids. When applying all features, 1.7287+E224 possible peptides could be generated, using tryptic digestion and allowing any number of missed cleavages and an arbitrary peptide length. This is a pure combinatorial approach, without biological or medical reasoning. In spite of the complexity of this protein-graph we demonstrate in Fig. 1 that it is possible to count the total number of peptides by any feature in reasonable time. Figure 1 shows the possible number of peptides for the respective allowed number of the feature variant per peptide. It also shows that by restricting the number of variants per peptide, the search space is reduced significantly, which can be generalized for each protein (see Supplemental Figs 4 and 5 showing further examples). With current hardware, it is necessary to limit the number of variants to e.g. 3–5 per peptide, especially if compute time or storage plays a crucial role. This also considers the fact that the occurrence of too many amino acid variations in a single peptide would lead to significant biological changes of the peptide, or, respectively, the protein.

**Figure 3:**
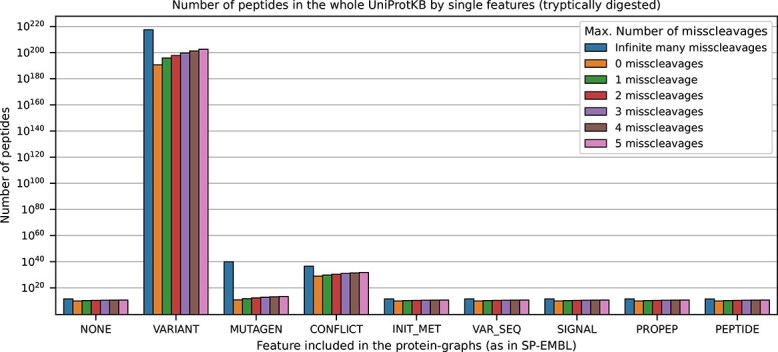
The total number of tryptic peptides in the whole UniProtKB, created by the respective annotated features. Applying the variant feature on the protein-graphs contributes to the highest total number of peptides, peaking at $1.7287+E224$. The features describing conflicts and mutagens on the protein-sequence contribute next in ranks to an increased number of total peptides. The highest peak in this figure is dominated by the human tumor protein P53 (see also Fig. 1).

Besides looking into a single protein-graph, we also counted the total number of possible peptides generated for different numbers of allowed miscleavage sites on the whole UniProtKB, applying either none or each kind of feature separately on the protein-graphs. Here, it can be observed that the variant-feature contributes to the highest amount of peptides within protein-graphs (Fig. 3), while all other features contribute significantly less to the search space.

Supplemental Table 2 gives insights about the canonical tryptic search space from a different perspective, e.g. showing that the total number of possible peptides increases when allowing more missed cleavage sites. These characteristics can be extracted from the protein-graphs within seconds and thus it can be decided beforehand whether an export into FASTA format for the spectrum identification is reasonable, or which respective parameters need to be applied.

### Spectrum matching using ProtGraph feature information

First we analysed the results of the workflow depicted in Supplemental Fig. 2a with parameters to only export canonical tryptic subsequences. As expected, this workflow yields nearly exactly the same identification results as the common workflow. Minor discrepancies can be seen in the PSMs expectation value, due to different numbers of entries in the sequence database, as well as rare differences in the creation of decoy sequences (due to different handling of cleavage sites).

The workflow depicted in Supplemental Fig. 2b (precursor specific workflow) was configured to also export annotated feature information, including up to five variant features per peptide. Detailed parameters can be found in the Methods section. These workflows, including additional steps for a complete benchmark respective proteomics identification analysis, are publicly available under workflowHub [20] and https://github.com/mpc-bioinformatics/ProGFASTAGen/. Supplemental Table 3 shows the respective execution times of all workflows.

**Figure 4:**
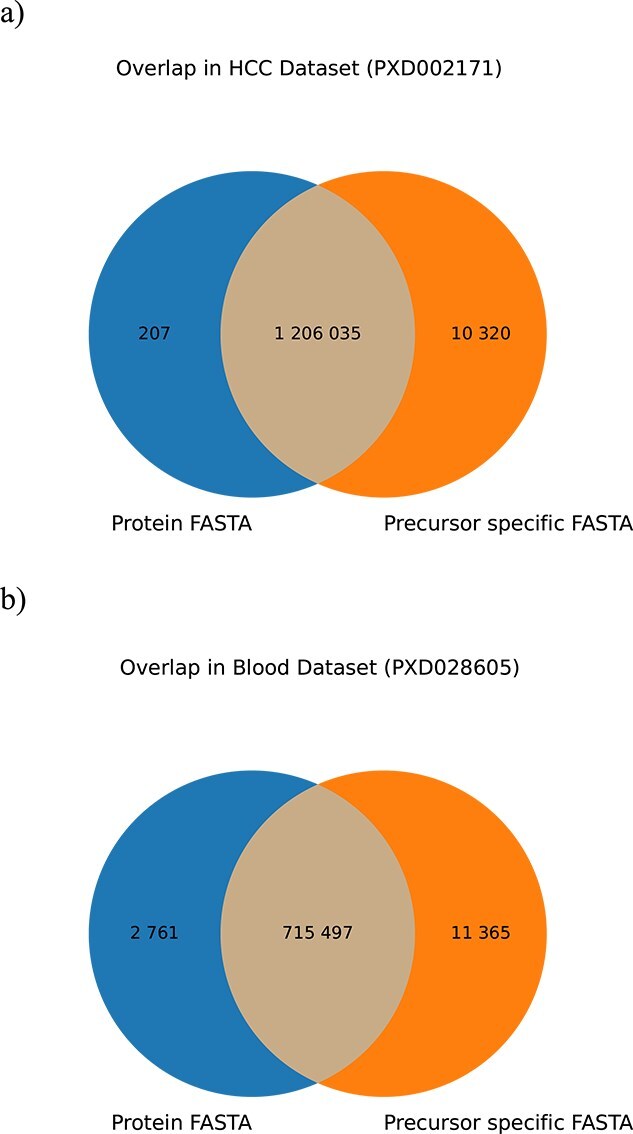
Venn-Diagram comparing the common workflow and the precursor specific workflow for PXD002171 (a) and PXD028605 (b) showing the overlap of spectra which received a peptide annotation (without FDR-estimation and filtering). It shows that some spectra only receive a peptide annotation by a specific workflow. The additional annotated spectra of the precursor specific workflow originate from peptides incorporating features, e.g. variant information, not present in common FASTAs. The vast majority of the spectra in the exclusive groups for both databases would not survive an FDR-threshold of 1%.

We further examined the PSMs returned from each workflow. First, it was analysed, whether spectra were matched against the feature-containing peptides in the precursor specific workflow. The venn diagram in Fig. 4 compares the spectra which received a peptide annotation across the two workflows, for this visualization without FDR estimation and filtering. Some spectra only received a match in the common workflow. This small difference can be explained by very small numeric differences in the calculation of the precursor error-tolerance boundaries of Comet and our workflow. However, with the precursor specific workflow many spectra, which would not get a peptide annotation by the common workflow, receive peptide annotations. If applying a q-value cutoff to these additional PSMs, only one spectrum in the HCC dataset and six spectra in the blood dataset would remain. These PSMs originate from peptides containing at least one feature annotation, which otherwise cannot be matched with a common FASTA.

**Figure 5:**
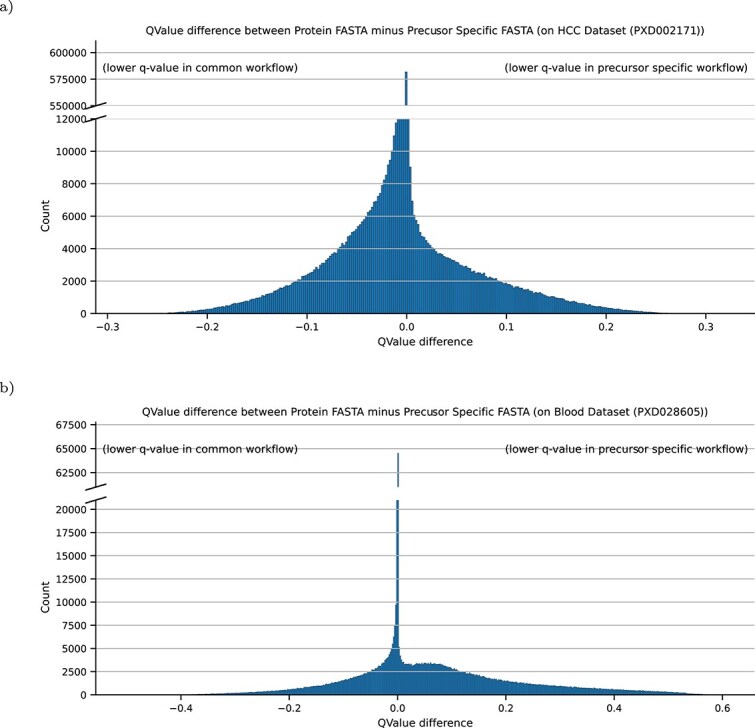
Q-value difference between same spectra across the common and precursor specific workflow for the HCC dataset (a) and blood dataset (b). In (a) a tendency towards the common workflow can be observed, indicating that the same spectra within the common workflow have lower q-values than in the precursor specific workflow. However, in the blood dataset (b), many spectra received better identification with the precursor specific workflow yielding lower q-values, showing a larger positive area in the differences. However most of the better matching spectra do not survive the q-value cutoff. If filtering for 1% FDR valid PSMs, most of the remaining entries are around a q-value difference of 0.

Furthermore, we inspected the annotated peptides of the same spectra between the common and precursor specific workflow and their respective q-value differences. These were visualized in the histograms of Fig. 5. It can be observed that most spectra have no or a very slight change in the q-value and thus have a difference close to zero. Smaller differences indicate a slight change in the ranking of the sorted PSM-lists, while larger differences may result from a better suited peptide-candidate, which is present only in the FASTA databases by the precursor specific workflow. A larger FASTA database leads to an increased number of estimated false positives [25] and this fact can also be observed in Fig. 5a, where more PSMs with a lower q-value are present in the common workflow. For the dataset PXD028605 (Fig. 5b), a second shoulder can be observed, indicating that for many spectra, when searching with a precursor-specific-FASTA, a better peptide-candidate has been selected. If applying a q-value threshold of 1% before calculation of the q-value differences, most entries around $.0$ remain for both datasets. The shoulder visible for the differences in the blood dataset cannot be observed in the filtered data, but it becomes obvious that many PSMs filtered out in the common workflow get an identification below the 1% q-value threshold with the precursor-specific workflow. This is depicted also in Supplemental Fig. 6.

### Workflow identification results considering q-value

**Figure 6:**
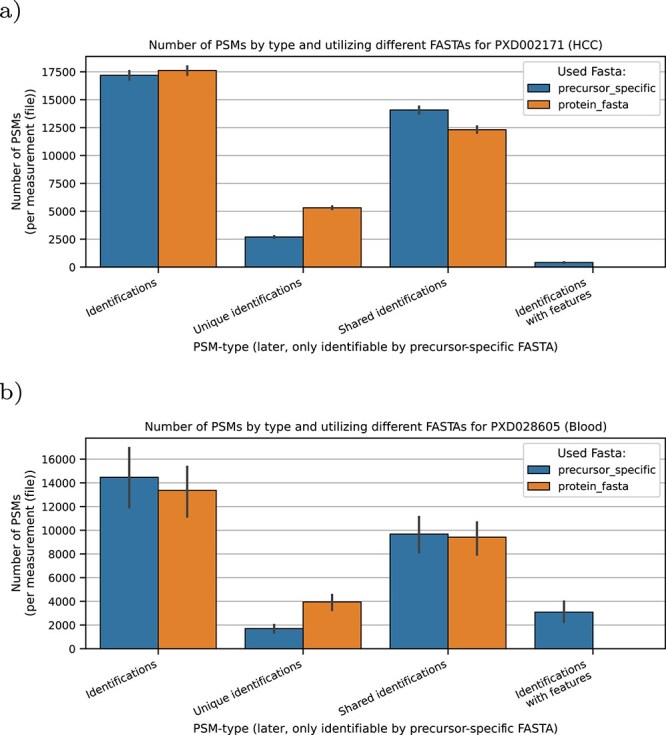
Number of FDR valid PSMs by the different workflows. Using the precursor specific workflow for the the HCC dataset yields less PSMS, while on the blood dataset more PSMs can be observed. The total number of unique PSMs decreases when a precursor specific workflow is used. For both datasets, spectra exclusively matching to feature peptides have been identified. In the blood dataset, they sum up to 23.1% of PSMs in average per run and for the HCC dataset to 2.4%.

We filtered the PSM results of the prior workflows using a q-value of 1% resulting in 17 000 to 17 600 PSMs per MS run for the HCC dataset and 13 000 to 14 000 PSMs for the blood dataset. Figure 6 gives an overview of the number of FDR valid PSMs. In general, we observed a decrease in the number of valid PSMs in the HCC dataset (Fig. 6a), but an increase in the blood dataset (Fig. 6b) when comparing the common and our precursor specific workflow.

**Figure 7:**
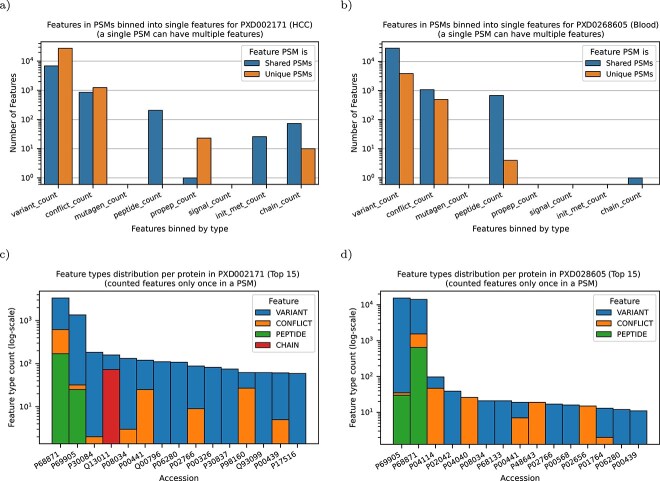
The number of found feature PSMs in FDR valid PSMs by their type, depicting results of all MS-Runs in the datasets in log-scale [(a) and (b)] and the top 15 proteins with identified unique feature PSMs [(c) and (d)]. On the left [(a) and (c)] for the HCC dataset and on the right [(b) and (d)] the blood dataset is visualized. From unique feature PSMs, the found peptide can only be explained by the used feature(s). On shared feature PSMs, it is inconclusive, since multiple and different features could result to the peptide match [(a) and (b)]. In (c) and (d), most prominently P68871 (HBB) and P69905 (HBA) have the highest number of found unique features, mostly consisting of variants.

We observed that 2.4% of the identified PSMs in the HCC dataset and 23.1% of the identified PSMs in the blood dataset could exclusively be associated with feature peptides from the precursor specific workflow and hence not identified by the common workflow. Notably, the number of shared peptides, i.e. peptides which can be mapped to more than one protein in the database, increases slightly when searching with the precursor specific workflow. This can be explained mainly due to the inclusion of isoform sequences. We further visualized the occurrences of feature types (excluding the isoform feature information, which is the only one which can be directly downloaded and used by the common workflow) in Fig. 7a and b. As expected from the distribution shown in Fig. 3, for both datasets mostly variant-features were identified, followed by conflict-features.

Further we examined by how many unique features the proteins were identified and which had the highest number of PSMs originating from these unique features (Fig. 7). In both datasets, common blood proteins (especially HBB (P68871) and HBA (P9905)) have the most associated variant features. This aligns with the fact that these proteins have next to P53 the most annotated variants in UniProt KB and highlights the importance of using these annotations for identifications. Although for HBA and HBB we found one order of magnitude more variants then in all other proteins, we still find many variants in the other less annotated proteins.

We further examined which actual sequence variants could be identified and counted the number of unique PSMs for these in both datasets. The results of the 15 most found sequence variants are displayed in Table 1, revealing that many variants co-occur simultaneously for HBB (P68871) and HBA (P69905). Of the shown data, there is with current knowledge only one variant associated with a disease. This variant feature describes a missing Valine at position 63 for the protein HBA (VAR_066401), which is associated with a form of alpha-thalassemia [Hemoglobin H disease (HBH)] [26]. In the other dataset this variant was also found, but only at position 19 and with two other variants co-occurring. Furthermore, in the HCC dataset the variant feature replacing Lysine with Valine at position 145 in the protein AKR1C4 (P17516, VAR_013291) was identified, which leads to decreased activity for xenobiotic and steroidal substrates [27], but only if found with another variant (VAR_013291) in combination. In our results, we did not encounter the other variant. Another interesting identified variant is VAR_022093, which replaces Threonine with Alanine at position 94 in the protein FABP1 (P07148), which may lead to an increased binding of cholesterol and possibly to several other effects [28].

**Table 1:** Top 15 actual sequence variants, counted by the number of unique FDR valid PSMs

Rank	HCC dataset		Blood dataset	
	#PSMs	Protein, co-occurring variant	#PSMs	Protein, co-occurring variants
1	233	HBB (P68871) VAR_002901 VAR_002904	541	HBB (P68871) VAR_002933 VAR_002934 VAR_002939 VAR_002941
2	175	ECHS1 (P30084) VAR_022274	427	HBA (P69905) VAR_002788 VAR_002795 VAR_002799
3	168	HBA (P69905) VAR_002788 VAR_002792	364	HBA (P69905) VAR_002788 VAR_002792
4	161	HBB (P68871) VAR_003077	335	HBB (P68871) VAR_002935 VAR_002937 VAR_002939
5	144	HBB (P68871) VAR_002922 VAR_035237	332	HBB (P68871) VAR_003077
6	106	SORD (Q00796) VAR_060351	236	HBA (P69905) VAR_002783 VAR_002789
7	86	ECH1 (Q13011) VAR_014927	235	HBA (P69905) VAR_002793 VAR_012662
8	82	HBA (P69905) VAR_002799 VAR_002808 VAR_002810	198	HBA (P69905) VAR_066401
9	82	ADH1C (P00326) VAR_000429	181	HBB (P68871) VAR_002923 VAR_002926 VAR_002927
10	77	HBB (P68871) VAR_002923 VAR_002926 VAR_002927	171	HBB (P68871) VAR_002923 VAR_002926 VAR_002927 VAR_002930
11	77	HBA (P69905) VAR_002793 VAR_012662 VAR_002802	168	HBA (P69905) VAR_066401 VAR_002792 VAR_012662
12	59	HBB (P68871) VAR_002933 VAR_002939 VAR_002944	165	HBB (P68871) VAR_035237
13	59	AKR1C4 (P17516) VAR_013291	144	HBB (P68871) VAR_035237 VAR_002933 VAR_002935 VAR_002939 VAR_002940
14	51	FABP1 (P07148) VAR_022093	143	HBA (P69905) VAR_066401 VAR_002788 VAR_002793 VAR_002795
15	50	SERPINA1 (P01009) VAR_007010	138	HBB (P68871) VAR_040060 VAR_002970

Most prominently, HBB (P68871) and HBA (P69905) have the highest number of PSMs identifying variants. Interesting variants linked to the clinical symptoms ‘increase of the binding for cholesterol’ (HCC dataset) and ‘Hemoglobin H disease’ (blood dataset) are highlighted

## Discussion

We showed that our approach of generating protein-graphs can be performed quickly, allowing arbitrary inclusion of annotated protein features and cleavage methods. For every protein in the UniProtKB, a protein-graph containing all current annotations could be generated and all possible peptides with their respective features, like sequence variants, could be exported into a FASTA. The applied optimization steps keep the protein-graphs compact and reduce the number of expanding steps during traversal, indirectly enhancing the performance of such algorithms. With the traversal time limitation described in the supplementary data section 1.7, we ensure that the sophisticated traversal is terminated in finite time for a quick precursor-specific FASTA generation. This approach adaptively limits the number of variants per peptide and thus allows FASTA exports of complex protein graphs derived from highly annotated protein entries, like P53 (P04637).

With the protein-graph retrievable characteristics of protein-graphs, information about search spaces from different perspectives can be generated. Such information can be helpful in the decision making of new proteomics related software and may be beneficial to enhance existing implementations, like in database search engines. For example, the characteristics about the number of possible tryptic peptides (Supplemental Table 2) in the whole UniProtKB limited by e.g. up to two miscleavages shows that approaches to store all peptides in databases like in [29] are feasible. Also new spectrum search engines could calculate their time requirements to search species-unrestricted or -restricted databases based on these numbers.

With the capability of quickly querying the protein-graphs for peptides of certain masses, we created a workflow exporting FASTA files for specific mass spectrometry based proteomic runs in a feasible run time. With this, we enable the proteomics community to perform protein identifications considering feature induced peptides, which are already annotated by the UniProtKB, but are currently rarely used. We showcased this possibility with Comet and Percolator, but other search engines such as Mascot and MS-GF+ were also successfully tested (data not shown). This flexibility allows the use of protein-graphs and their generated FASTA exports to be integrated into well established workflows, giving researchers the opportunity to search for peptides annotated with features. Additionally to the annotated features in UniProtKB, in general any annotation e.g. derived by transcriptomics or genomics data can be incorporated into canonical protein sequences. Thus, a sample specific database containing specific variants can easily be created with ProtGraph, benefiting the fields of proteogenomics and metaproteomics.

For two analysed dataset s, we demonstrated that peptides with annotated features can be identified, providing new details and perspectives for a more in-depth analysis. Although the total number of identifications decreases in the HCC dataset by using a precursor specific FASTA, more precursors are assigned to peptides as to the protein FASTA, yielding more PSMs before the q-value cutoff as shown in Fig. 4. However, this gain of PSMs comes with a caveat: the more entries a FASTA-database contains, the less PSMs survive the q-value cutoff. This effect, also known as FDR-Overestimation, is observable between a protein and precursor specific FASTA as seen in Figs 5 and 6a for the precursor specific FASTA, where less PSMs are present. Recently it was shown that more modern rescoring methods, employing machine learning, such as MS2Rescore [30–32] can counterfeit the drop of identified PSMs with large FASTA databases. While this drop can be observed for the HCC dataset, we see an increase of filtered PSMs for the blood dataset, due to a better fit of the precursor specific FASTA database, further pinpointing that FASTA databases heavily influence identification results. Nevertheless for both datasets it can be beneficial to search for peptides with annotated features. As observed in both datasets, it is expected to have a lot of variation in blood proteins, since these are also highly annotated.

Our results include hits which are linked to HBH disease, describing a form of alpha-thalassamia that leads to reduced hemoglobin production in the body. In another study by Zhang et al. [33], low pre-treatment hemoglobin levels were shown to be an independent biomarker for the poor prognosis for NSCLC. While the workflow only provides identification information, we demonstrate that this variant may be present in both groups and showcase the increase of meaningful identifications with the precursor specific workflow, which could be interesting biomarker candidates.

## Conclusions

While we showed that the application of ProtGraph enables the identification of variants and other features for arbitrary datasets, it still is more time consuming than the common workflow. Depending on the research question and the knowledge whether the analysed samples potentially contain variants, it is preferable to invest this additional resources and time, to get a more complete and comprehensive analysis.

Protein graphs can also be applied to the following use-cases: graph visualizations (see Fig. 2) can provide a human-interpretable graphical illustration of features around peptide sequences, similar to protein association networks (e.g. String [34]). Furthermore, this approach could be used as a mapping between identification generated by de-novo-sequencing methods (like PepNovo [35]) and protein entries with annotated features provided by UniProt. Additionally, it may be ideal to incorporate protein-graphs directly into search engines without the need for a FASTA and even match them to so-called spectrum graphs, similar to the approach in the MS-GF+ [7] search engine.

To our knowledge ProtGraph is the first tool in the proteomics field which can map all annotated sequence features of UniProtKB into a compact structure. Furthermore, it allows the use of precursor specific FASTA databases in already available spectrum search engines without any further modifications and thus uses this additional information for spectrum identification. Therefore, ProtGraph enables to incorporate the vast annotation information stored in UniProtKB, which until now is almost never used by the community, or other sources, derived e.g. from genomics or patient specific transcriptomes. By using the generated precursor specific FASTA, it enables the identification of such features on arbitrary search engines and workflows.

**Key Points**

- ProtGraph allows the concise and comprehensive representation of protein sequences including annotated variants and other features in a graph structure.
- This enables the fast extraction of all possible peptides created by the protein graphs using different filters, e.g. for masses of MS/MS precursor ions or specific peptide lengths.
- ProtGraph also enables a very quick and comprehensive characterization of the number and types of annotated variants and other features occurring from e.g. protein maturation.
- We created workflows for the re-analysis of datasets by common proteomics search engines employing protein sequence databases with all annotated protein variants and features in UniProt and benchmarked these on two publicly available datasets, yielding hitherto overlooked insights into proteins with non-canonical sequences.

## Supplementary Material

ProtGraph_supplemental_bbae671

P04637_runtime_float_bbae671

P68871_runtime_float_bbae671

supplemental_bbae671

## Data Availability

Exact workflow execution can be found in https://github.com/mpc-bioinformatics/ProGFASTAGen/. Both re-analyses were uploaded to the ProteomeXchange Consortium (http://proteomecentral.proteomexchange.org) via the PRIDE partner repository [24] with the dataset identifier PXD053196 and DOI 10.6019/PXD053196 (HCC dataset) PXD053237 and DOI 10.6019/PXD053237 (blood dataset), respectively.
