# Correction to: ProtGraph: a tool for the quick and comprehensive exploration and exploitation of the peptide search space derived from protein sequence databases using graphs

**DOI:** 10.1093/bib/bbaf222

**Published:** 2025-05-06

This is a correction to: Dominik Lux, Katrin Marcus-Alic, Martin Eisenacher, Julian Uszkoreit, ProtGraph: a tool for the quick and comprehensive exploration and exploitation of the peptide search space derived from protein sequence databases using graphs, *Briefings in Bioinformatics*, Volume 26, Issue 1, January 2025, https://doi.org/10.1093/bib/bbae671

The following changes have been made to the originally published manuscript. Respective markup in the author byline has been made and a statement added regarding equal contribution made by two-co-authors to this paper: Martin Eisenacher and Julian Uszkoreit.

