# Supplementary material for: ProtGraph: a tool for the quick and comprehensive exploration and exploitation of the peptide search space derived from protein sequence databases using graphs
Source: Brief Bioinform. 2025 Jan 5;26(1):bbae671. doi: 10.1093/bib/bbae671 (PMC11700661; doi:10.1093/bib/bbae671)
Supplement: ProtGraph_supplemental_bbae671 [file protgraph_supplemental_bbae671.pdf]

ProtGraph: a tool for the quick and comprehensive exploration and exploitation of the peptide search space derived from protein sequence databases using graphs

Supplementary Material

Dominik Lux, Katrin Marcus-Alic, Martin Eisenacher, Julian Uszkoreit

1 Supplemental Methods

1.1 Extending the protein-graph with features introducing protein cleavages

The initial graph PG_i is first extended by features which are annotated as SIGNAL, PEPTIDE, PROPEP, CHAIN and INIT_MET. We specifically apply these features first to avoid ambiguity with enzymatic cleavage sites. Let C be the set of edges which would be cleavage sites in PG_i by a given feature. We then retrieve all vertices being the source (before) and the target (after) of the directed edges in C: $B := \{n | n \in V, e \in C, n == e.source\}$ and $A := \{n | n \in V, e \in C, n == e.target\}$ respectively.

To introduce a cleavage site into the graph, additional edges are added in order to map all valid paths. This can be achieved by introducing edges from the start node to A and from B to the end node. Explicitly we add the edges resulting from the cartesian product: $(B \times \{e\})$ and $(\{s\} \times A)$, adding in total $|B| + |A|$ many edges. Additionally, the original feature information is added to the qualifier-labels of all newly created edges. The INIT_MET feature is an exception to this rule, where only edges from the cartesian product $(\{s\} \times A)$ are added, since the solely cleaved Methionine is not regarded as peptide furthermore.

1.2 Extending the protein graphs with variational features

Next, features containing sequence variations are added into the graph. These features are annotated as VARIANT, MUTAGEN and CONFLICT in the UniProt flat file format entries.

Focusing on the feature entries, information about the original amino-acid chain (X) with its substitution (Y) information is provided and corresponding vertices in the protein-graph can be determined. For substituting X with Y, two cases need to be considered: A missing/empty annotation in Y (effectively cleaving X from the protein-graph) and a replacement with a chain Y. For both cases, all vertices before (B) and after (A) are determined first in the same manner as in the protein-cleavage step. We define an intermediate set M, containing the same ingoing edges as the first vertex of the chain X and vice versa for the last vertex of the chain X in the protein-graph and use this set to retrieve B and A.

For the first case, cleaving X from the protein-graph, edges from the cartesian product $(B \times A)$ are added. Additionally, to these newly added edges, the qualifier-label is appended to contain the feature information which has been applied.

In case of a non-missing/non-empty substitution of Y, a chain of vertices for each amino acid in Y is created, similar to the initial protein-graph PG_i. Let Y_s denote the beginning of the chain Y and Y_e the ending of the chain Y, to extend the protein-graph with the sequence variation, the edges resulting from the cartesian product $(B \times \{Y_s\})$ and $(\{Y_e\} \times A)$ are added and feature information are appended to the qualifier-label for all edges in $(B \times \{Y_s\})$ in order to assess from which feature this sequence variation was originating.

With this mechanism, variable post translational modifications can be handled in a similar way, since these can be also treated as a sequence variation. These then substitute the same amino-acid with itself and a mass shift (e.g. M substitutes to M+18). As relevant modifications can be specific to a peptide N- or C-terminus, these need to be added after the digestion step.

For amino-acid replacements (e.g. ambiguous amino acids like J $\rightarrow$ I and J $\rightarrow$ L) the same mechanism is applied, but the original amino-acid gets deleted from the protein-graph.

1.3 In-silico digestion of the protein-graph

The in-silico (enzymatic) digestion of a protein-graph works with the same ruleset as the introduction of features with cleavages, but needs to be introduced after the variational features. While digesting, the set C describes in this case all edges, which would mark the cleavage points of a digestion enzyme (e.g. Trypsin, but also other enzymes and unspecified cleavages at each amino acid are possible). No feature information is added to the qualifier-labels, instead all edges in C are marked with the label cleaved, which later is used to represent miscleavage sites. This special case allows the protein graph after the digestion to contain peptides of the original protein with up to arbitrarily many miscleavages.

1.4 Optimising protein-graph

After applying all features, digestion and PTM annotations on the protein-graph, we optimise for a compact protein-graph representation. This step aims to reduce the graph size by detecting uninterrupted chains of connected vertices and replacing them with a summarised vertex. To achieve this, the in- and out-degree of vertices are used to recognise chain parts in a protein-graph. Let $CB = \{n | n \in V, n.indegree > 1, c.outdegree = 1\}$, $CM = \{n | n \in V, n.indegree = 1, n.outdegree = 1\}$ and $CE = \{n | n \in V, n.outdegree > 1, c.indegree = 1\}$ be the set of vertices for a possible beginning, elongation and the end of a chain respectively. To finally retrieve the chain, the algorithm displayed in **Listing** 1 is applied.

Listing 1: Retrieve chained nodes

```
init
  chains = []

begin
  for n_cb in CB:
    path = [n_cb]
    while n_next :=
         path.end().edge[0].target ∈ (CM ∪ CE):
      // The vertex at the end in path can only contain 1 edge
```

```
      (CM ∪ CE).remove(n_next)
      path.append(n_next)
    chains.append(path)
  return chains
end
```

For the remaining vertices in the set CM, the same algorithm is applied a second time, iterating over CM, to further extend the list of chains. The second application covers special cases, where chains begin with a single ingoing edge (e.g. the beginning of a chain as a successive vertex after the start vertex s). For each chain that has been retrieved, a summarised vertex is generated, concatenating the information of the labels, like amino-acids, or using the label of the first vertex in the chain, like the position. Similar to the extension of variational protein-features in protein-graphs, the summarised vertex gets attached to the position of the chain in the protein-graph, copying the label information of the edges of the chain beginning and ending. Additionally, the original chain, with all its attached edges are removed, effectively replacing the chain with a single summarised vertex.

1.5 Retrieving the number of peptides contained within a protein-graphs

After a protein-graph has been generated from a protein entry, it opens several possibilities for characteristics to be calculated. We primarily focus on the number of possible peptides a protein-graph contains and therefore define the number of possible peptides as all peptides that are attainable via a valid path, i.e. starting in s and ending in e. Peptide sequences are counted multiple times, if there are multiple valid paths resulting in the same peptide sequence. The number of valid paths is therefore an upper limit of actual unique peptide sequences within a protein-graph.

To retrieve the number of possible peptides, the number of valid paths in a protein-graph is calculated by exploiting the topological order with a dynamic programming approach. The number of valid paths can be retrieved with the following implementation **Listing** 2:

Listing 2: Retrieve number of possible peptides

```
init
  // Get Protein-Graph Top. Order
  top_order = pg.getTopOrder()
  paths = map<vertex, value>
  paths[top_order[0]] = [1] // Initial Value for vertex s

begin
for v in top_order[1:]:
  result = [0] //  Initial intermediate Value
  for e_in in v.inedges():
    // Kernel
    If bin_feature(e_in): // E.G. Variant on Edge
      // Add leading 0 and add element wise
      result += ([0] + paths[e_in.source])
    else:
      result += paths[e_in.source] // Add element wise
    paths[v] = result
  // Results of the last vertex are only reported
  return paths[top_order[-1]]
end
```

This algorithm runs in worst case $\mathcal{O}(n^2)$ if "bin_feature(e)" always yields False (see section 3). If desired, this algorithm can further distinguish by the number of possible peptides, binned to a specific property, by exploiting the list structure (hence a runtime of $\mathcal{O}(n^3)$. E.G. If an edge contains a variant, the list can be shifted by a leading 0, binning the number of applied variants to list indices yielding the number of possible peptides binned by the number of applied variants (as shown in **Figure 1** in the manuscript or **Supplemental Figure** 5 or 5). This approach can be used to retrieve the number of peptides without binning or binned by miscleavages, variants, mutagens, conflicts or peptide lengths in protein-graphs.

1.6 Naïve traversal of protein-graphs to generate complete FASTA exports

Currently it is not possible to search directly for spectrum matches within the described protein-graphs using common peptide search engines. An export into a FASTA-file is therefore needed, to make the information in protein-graphs accessible to search engines. We use a depth-first search approach to traverse protein-graphs, by starting the traversal at the start vertex s and report every valid path ending in the vertex e. The expanding step to the next vertex of this depth-first search can be constrained by a maximal peptide length, peptide weight and number of missed cleavage sites. Additionally, lower limits can be set to disregard very short peptides. The valid and remaining paths are reported by this implementation and converted into FASTA entries (see section 2). The resulting FASTA file, containing all valid peptides of the protein graphs can be further processed to group the header information of identical sequences. Such a FASTA export can directly be used by common peptide search engines neglecting further in-silico digestion, as the entries are already given as the mandatory peptides.

1.7 Sophisticated traversal of protein-graphs to generate precursor specific FASTA files

The naïve traversal is only feasible if the number of peptides/proteins to export is relatively small, which can be checked prior via the methods described in **Listing** 2. To handle larger search spaces, a more sophisticated traversal algorithm has been developed, which extracts only valid paths, i.e. peptide sequences, from protein graphs, whose masses fit to a precursor of a MS2 spectrum. This reduces the size of the resulting FASTA file and allows it to contain only entries, which are needed by the search engines during identification. We use the target-value search described by Schmidt et al. [1] to quickly access possible masses inside the protein graph. The multi-interval pattern databases (PDBs) are built using the reverse topological order of a protein-graph and added to the vertex labels. Furthermore, we replaced the originally proposed traversal algorithm by Schmidt et al. and extended the query to a query interval as an input. The whole algorithm is described in **Listing** 3 and has a running time of $\mathcal{O}(2^{n-1})$ as shown in section 4.

Listing 3: Retrieve peptide sequence by mass from protein-graph

```
init
  // The input query as an interval [X, Y]
  lower, upper = [X, Y]
  // Number of features (e.g. variants allowed) per path
  feature_threshold = k
  // Achieved target values
```

```
  tv_entries = map<vertex, list<values>>
  // The corresponding paths to tv_entries
  paths = map<vertex, list<paths>>
  // Top. Order of a protein-graph
  top_order = pg.getTopOrder()
  tv_entries[top_order[0]] = [0] //Initial value
  paths[top_order[0]] = [0] // Initial value

begin
  for n in top_order: // For each node
    for e in n.outedges(): // For each outgoing edge
      // For each intermediately achieved path
      for tv_current, p in tv_entries[n], paths[n]:
        tv_achieved = tv_current + e.target().peptide_weight
        // Check each interval at target node
        if overlapping_interval(
          e.target(), lower - tv_achieved, upper - tv_achieved
        ) && count_features(p) < feature_threshold:
          // Case: Any interval of the target node overlaps with
          // the queried interval and has less then
          // feature_threshold features → expand
          tv_entries[e.target()].append(tv_achieved)
          paths[e.target()].append(p.append(e.target()))
  // Return all paths leading to the end node fitting to the query
  return paths[top_order[-1]]
end
```

The input interval query can be generated by calculating the precursor tolerance window. This is usually denoted as ppm or in Dalton (Da), and is a common parameter of the mass spectrometer, which is also needed by peptide search engines. An additional threshold can be specified, allowing a maximum number of variants (or features) per exported sequence path, restricting the traversal further. This algorithm needs to be executed once per precursor tolerance window, i.e. roughly per measured peptide in a sample, for every protein-graph (i.e. queried protein). Since protein-graphs may contain an arbitrary number of valid paths, which grows exponentially with the number of features it holds, this algorithm may not terminate in feasible time for highly complex protein-graphs on specific queries.

To allow an export of these complex protein-graphs in a feasible amount of time, we implemented a binary-search around **Listing** 3 which determines a maximum threshold of allowed variants (or features) per query interval of each protein-graph, which can be processed in a given timespan. The algorithm needs two parameters to determine the limits of allowed variations: A timeout (in seconds), which gives the search algorithm an upper runtime limit and the number of mass bins of the protein-graph exports. The timeout parameter is not the actual time needed for the export of results with the respective maximum mass and Dalton, which might be higher, but the time the protein-graph is traversed with the given parameters. To smooth the runtime results, a 3-window median filter was applied after determining the thresholds to ensure algorithm-termination. **Supplemental Figure** 1 exemplary shows the results after the binary search on P53 (P04637). It can be seen that lower masses can be queried with more allowed variations in a reasonable time, while higher masses need exponentially more time to be processed with the same number of variants, due to the exponential

possible combinations of variants. But it is also shown that this growth can be sufficiently limited by allowing fewer variations per peptide.

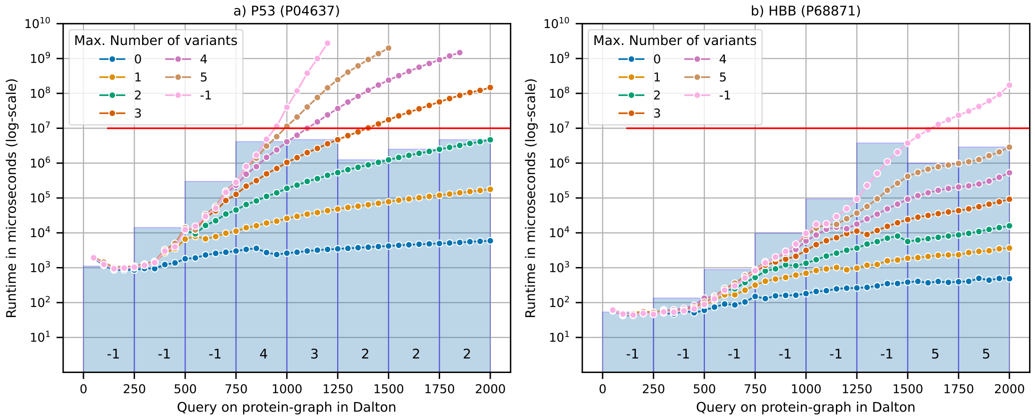

Figure 1: Running time in microseconds (log-scale) plotted against the queried mass in Dalton (top) and visualized bins (of size 250 Da) with selected variant-limits after the binary search. For the query, a 5ppm tolerance was set, while restricting the number of variants. In a) and b) the query time was measured for every 50 Da, up to 2000 Da. For P53 (P04637, a)), it can be observed that the running time is decreases drastically when fewer variants are allowed per peptide, restricting the search space significantly. In HBB (P68871, b)), a similar reduction can be observed. A red line illustrates the set timeout for the algorithm (here 10 seconds). After the binary search on each bin, maximum variant limits are determined (labeled on each bin) showing that the higher the query is, the less variants will be used for the query. Raw runtime numbers can be found in the **supplementary data**.

The proposed algorithms allow to export as many combinations of variations as are feasible by a given runtime, instead of making a general restriction for all proteins. As this is highly dependent on the actual hardware, the possible amount of combinations will increase with faster hardware in the future. An exported FASTA file can be treated in the same way as the direct export described above.

2 Custom FASTA-Header and Sequence Uniqueness Definition

The resulting FASTA-databases, which are generated from the workflows using ProtGraph have a custom FASTA-header, encoding various information along a valid path. The custom FASTA headers follow the structure from the UniProtKB [7] FASTA headers, dividing the header in 3 essential parts: database-description, accession and description. For the database-description we set ”>pg”, to indicate that these entries were generated from a protein-graph. The accession has been repurposed and an unique identifier is set, which allows a mapping to the original FASTA-entry and its description for all PSMs for arbitrary search engines. The description contains at least 1 Protein, originating from this peptide, using the UniProtKB-accession, and in parenthesis, information about the position number of miscleavages and annotated features, which were retrieved along a path from a protein-

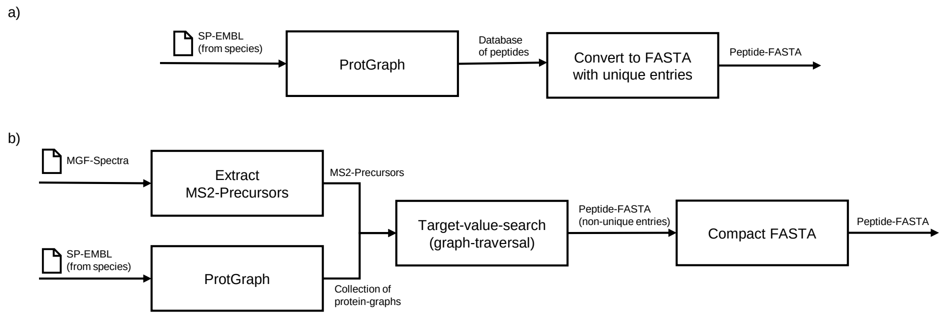

Figure 2: Illustration of the implemented workflows of how protein-graphs can be used to generate FASTA databases. In a) a workflow for a global-peptide-FASTA-database, which can be limited by global parameters is visualized. b) shows a workflow utilizing the MS2-precursor masses of the measured MS2-spectra, querying the generated protein-graphs using the traversal algorithm described in the methods section to only save peptides fitting to the precursor. Both workflows have been implemented individually in nextflow (and can be found in https://github.com/mpc-bioinformatics/ProGFASTAGen/.

graph. By the number of protein accessions in the description, it can be deducted if a peptide is shared by one or more proteins. A peptide-sequence is therefore a unique sequence, if the description in the FASTA contains only a single protein, in other words, the sequence can only be mapped to a single protein in the selected protein-database. Furthermore, we define an entry as a unique feature peptide, if it can only be generated by a feature and is not in the database otherwise. Shared feature peptides are always described by annotated features from multiple proteins and are not present in the canonical database.

3 Run Time and Memory Usage of retrieving the number of peptides/proteins within protein-graphs

First we do the run time analysis for the algorithm presented in **Listing 2**. The worst protein graph structure as an input for this algorithm, would be a graph where every possible path combinations needs to be summed up, leading to a fully connected directed acyclic graph. A graph, where its first node (in a topological order) would have a directed edge to every other node, in total $n-1$ outgoing edges, the second node $n-2$ outgoing edges, etc. An example of such a graph is illustrated in Figure 3, representing for n nodes, 2^n-1 paths. The algorithm in **Listing 2** can also be applied generally on directed and acyclic graphs and is not limited only on protein graphs.

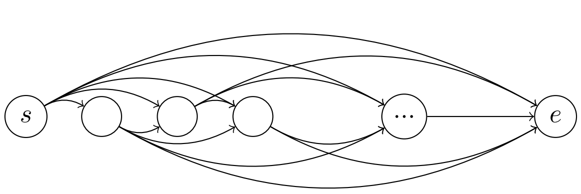

Figure 3: Fully connected directed acyclic graph. Each node can reach every successive node. This is a worst-case direct and acyclic (protein) graph. The start and end node are illustrated as an example in this graph.

3.1 Case: Without Binning

We need to distinguish between two cases in the algorithm in **Listing 2**. First we retrieve the runtime where no features are binned. For each node, which is already provided in the topological order, we have to check $n-1$, then $n-2$, ... 2, 1 and 0 edges. This can be expressed in the following equation, where k is the current iteration in the loop and n is the number of nodes in the graph:

$$\sum_{k=1}^{n} n-k \tag{1}$$

This equation can be rearranged and the complexity can be read out directly:

$$\mathcal{O}(\sum_{k=1}^{n} n-k) = \mathcal{O}(\frac{n^2}{2} - \frac{n}{2}) \approx \mathcal{O}(n^2) \tag{2}$$

This fits to the run time of dynamic programming approaches and shows, that the number of paths is retrievable from arbitrary graphs in at most quadratic running time. The memory usage for the such graphs is $\mathcal{O}(n)$ (linear), since each node saves an intermediate result of how many paths to itself are possible.

3.2 Case: With Binning

In case that the binning evaluates in every iteration to true for the algorithm **Listing 2**, the element-wise addition of two vectors need to be considered. In the first iteration at the first node, the vectors contain only a single element and only adds 1-many extra operations for $n-1$ many edges. However, on the second iteration, where now $n-2$-many edges need to be checked, the vectors already contain two elements, adding 2 extra operations, increasing by 1 for each iteration until to the last iteration, where the vector contains n-many entries. Let again n be the number of nodes and k the number of

iterations, the number of operations can be expressed as:

$$\begin{aligned}
\sum_{k=1}^{n}(n-k)\cdot k &= \sum_{k=1}^{n} nk - \sum_{k=1}^{n} k^2 \\
&= n \cdot \sum_{k=1}^{n} k - \sum_{k=1}^{n} k^2 \\
&= n \cdot (\frac{n\cdot(n+1)}{2}) - (\frac{n\cdot(n+1)\cdot(2n+1)}{6}) \\
&= \frac{3n^2\cdot(n+1)}{6} - \frac{n\cdot(n+1)\cdot(2n+1)}{6} \\
&= \frac{3n^3+3n^2}{6} - \frac{2n^3+3n^2+n}{6} \\
&= \frac{n^3-n}{6}
\end{aligned} \tag{3}$$

Thus, the run time complexity for binning at every iteration is $\mathcal{O}(\frac{n^3-n}{6}) \approx \mathcal{O}(n^3)$. For the memory usage, we can see that the vector increases in each iteration at most by 1. Similar to equation 1, we can use the same equation to describe the memory usage. Therefore, the highest memory consumption would be $\mathcal{O}(n^2)$.

4 Run Time and Memory Consumption for Retrieving interval fitting Paths

The algorithm in **Listing 3** checks for every path, if the sum of all node's (or edge's) weight in the path lies inside a given interval and reports those. In general, this could be the weights of amino acids, or the path length itself in other use cases. The directed graph in figure 3 is also for this algorithm the worst case, since it contains the maximum number of paths (2^{n-1}) which are possible result candidates. Pre-calculated Intervals per nodes, where minimal and maximal attainable partial paths are described, are used as an early detection of unfitting paths and to prevent expansions and to increase the efficiency. However, to force checks for every possible path, we can query for paths fitting to the interval: $[0, \infty]$. In other words, we want to retrieve all possible paths from a directed acyclic graph. With, the worst case and the large query, we can visualize the number of operations for each iteration of the outer loop in the table 1:

Iteration	Edges to Check	Paths to Check	Intervals to Check
1	$n-1$	1	1
2	$n-2$	$1=2^0$	1
3	$n-3$	$2=2^1$	1
4	$n-4$	$4=2^2$	1
...	...	...	...
$n-2$	2	2^{n-4}	1
$n-1$	1	2^{n-3}	1
n	0	2^{n-2}	1

Table 1: Illustration of operations for each iteration for the outer loop.

Similar as to the algorithm in **Listing 2**, the second loop needs to check for every edge, which decreases by one after each iteration. The third loop in **Listing 3** checks for each path, if it needs to be expanded, since it may lay in the final set of solutions. In this worst case, every path is expanded, therefore the number of paths increase exponentially after each step. However, for the first iteration, the first node is initialized with a single path, which is an exception to the exponential increasing number of paths, hence the shifting by 1. Finally the last loop iterates over each pre-calculated interval, which is saved on each node individually. Here, the algorithm yields true on the first pre-calculated interval and is the best case, since any arbitrary positive interval is also intersecting with $[0, \infty]$. Summarized with table 1, the number of operations can be expressed by the following equation:

$$\begin{aligned}(n-1)+\sum_{k=2}^{n}(n-k)\cdot 2^{k-2} &= (n-1)+\sum_{k=0}^{n-2}(n-k-2)\cdot 2^{k} \\ &= (n-1)+2^{n+1}+2^{n-1}+\sum_{k=0}^{n}(n-k-2)\cdot 2^{k}\end{aligned} \tag{4}$$

The sum has been re-indexed to contain $k=0$ and n as the lower and upper bound of the sum for further steps. The sum itself can also be rearranged into three simpler sums:

$$\sum_{k=0}^{n}(n-k-2)\cdot 2^{k} = n\cdot\sum_{k=0}^{n}2^{k}-\sum_{k=0}^{n}k\cdot 2^{k}-2\cdot\sum_{k=0}^{n}2^{k} \tag{5}$$

The first sum of the former result can be rearranged with a higher exponent, while the other sum is a finite geometric series, which can be rewritten as shown in equation 6.

$$\sum_{k=0}^{n}2^{k}=2^{n+1}-1 \qquad\qquad \sum_{k=0}^{n}k\cdot 2^{k}=2\cdot(n\cdot 2^{n}-2^{n}+1) \tag{6}$$

Using the knowledge of equation 6, the sum can be rearranged to the following:

$$\begin{aligned}\sum_{k=0}^{n}(n-k-2)\cdot 2^{k} &= n\cdot\sum_{k=0}^{n}2^{k}-\sum_{k=0}^{n}k\cdot 2^{k}-2\cdot\sum_{k=0}^{n}2^{k} \\ &= (n\cdot(2^{n+1}-1))-(2\cdot(n\cdot 2^{n}-2^{n}+1))-(2\cdot(2^{n+1}-1)) \\ &= (n\cdot 2^{n+1}-n)-(n\cdot 2^{n+1}-2^{n+1}+2)-(2^{n+2}-2) \\ &= -2^{n+2}+2^{n+1}-n\end{aligned} \tag{7}$$

And inserting equation 7 to the original equation, yields the run time for the algorithm in **Listing 3**

$$
\begin{aligned}
(n-1) + \sum_{k=2}^{n}(n-k)\cdot 2^{k-2} &= (n-1) + 2^{n+1} + 2^{n-1} + \sum_{k=0}^{n}(n-k-2)\cdot 2^{k} \\
&= n-1+2^{n+1}+2^{n-1}-2^{n+2}+2^{n+1}-n \\
&= n-1+2\cdot 2^{n+1}+2^{n-1}-2^{n+2}-n \\
&= n-1+2^{n+2}+2^{n-1}-2^{n+2}-n \\
&= n-1+2^{n-1}-n \\
&= 2^{n-1}-1 \\
&\approx \mathcal{O}(2^{n-1})
\end{aligned}
\tag{8}
$$

This shows that the run time for the algorithm in **Listing 3** is exponential. Regarding the memory usage, it can be seen at the return value in the algorithm in **Listing 3**. After iterating through the graph, the algorithm needs to save at the last node 2^{n-1} paths which it would then report, therefore occupying $\mathcal{O}(2^{n-1})$ space.

5 Additional Figures

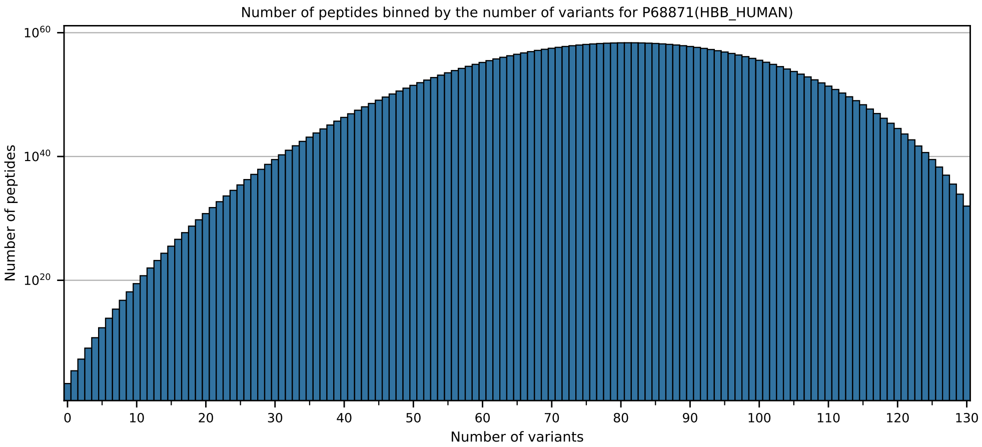

Figure 4: Total distribution of peptides binned by the number of variants per peptide for P68871 (HBB_HUMAN). For additional information compare **Figure 1**

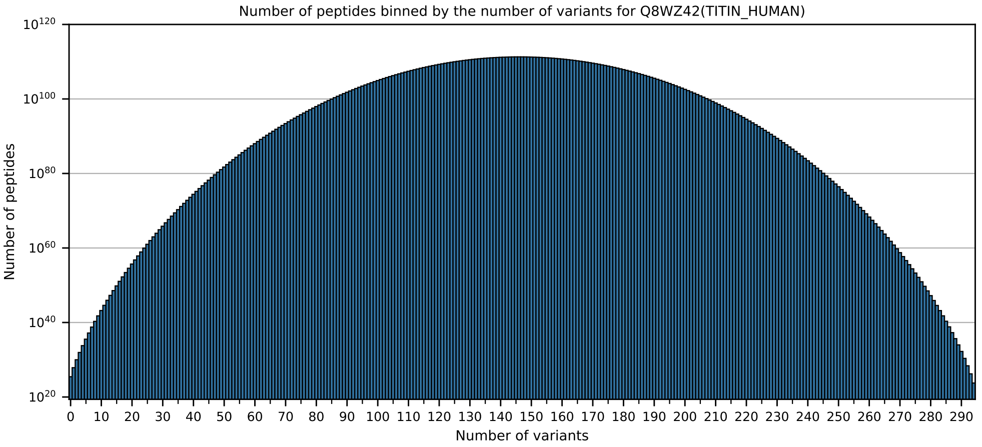

Figure 5: Total distribution of peptides binned by the number of variants per peptide for Q8WZ42 (TITIN_HUMAN). For additional information compare **Figure 1**

a)

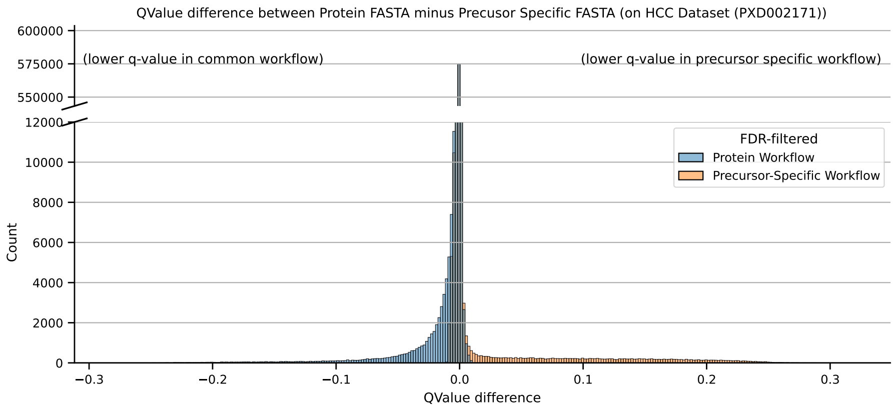

b)

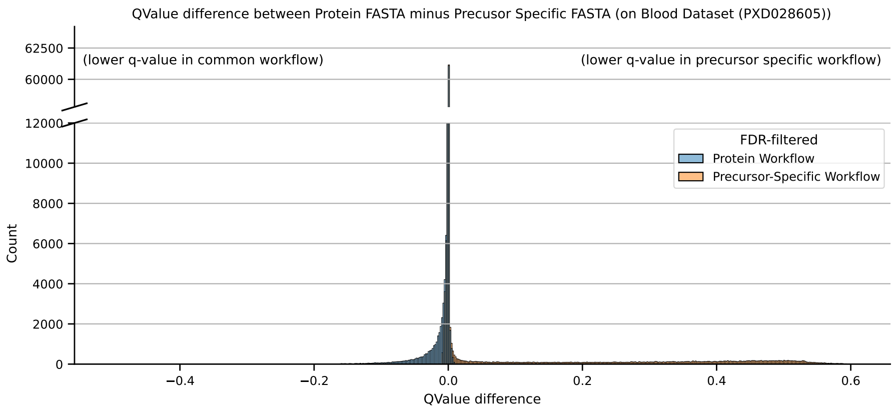

Figure 6: Qvalue difference between same spectra across the common and precursor specific workflow for the HCC dataset a) and Blood dataset b) for only 1% filtered peptide spectrum matches. It can be observed that many filtered peptide spectrum matches, which received a better peptide candidate through the precursor specific workflow remain.

Table 2: [a) and b)] The total number of peptides contained in the UniProtKB, without any sequence variations. The statistics were generated by converting each protein into a protein-graph, while applying no additional feature but only in-silico digested with Trypsin and allowing the given number of missed cleavage sites a) or peptide length b). This table therefore represents the tryptic search space of the canonical sequences.

a)

#misscleavages	#peptides (cummulative)	#peptides (exact)
0	9 011 818 663	9 011 818 663
1	17 779 342 593	8 767 523 930
2	26 302 854 601	8 523 512 008
3	34 583 077 755	8 280 223 154
4	42 621 185 830	8 038 108 075
5	50 419 119 874	7 797 934 044
6	57 979 722 074	7 560 602 200
7	65 306 240 285	7 326 518 211
8	72 402 164 165	7 095 923 880
9	79 271 412 345	6 869 248 180
10	85 918 318 292	6 646 305 947
...	...	...
59	242 043 370 986	1 380 768 047
60	243 388 708 778	1 345 337 792

b)

#misscleavages	#peptides (cummulative)	#peptides (exact)
0	0	0
1	1 157 469 714	1 157 469 714
2	2 251 365 132	1 093 895 418
3	3 340 963 267	1 089 598 135
4	4 406 278 517	1 065 315 250
5	5 457 218 194	1 050 939 677
6	6 480 734 353	1 023 516 159
7	7 529 077 636	1 048 343 283
8	8 557 850 058	1 028 772 422
9	9 552 878 527	995 028 469
10	10 542 144 593	989 266 066
...	...	...
59	54 739 705 443	830 513 073
60	55 570 637 303	830 931 860

Table 3: Overview of the running time (and number of entries in FASTA) for both datasets across different workflows. Generating a precursor specific FASTA consumes most of the execution time and can differ depending on the input dataset.

	HCC dataset			Blood dataset		
	common workflow	global workflow	precursor specific workflow	common workflow	global workflow	precursor specific workflow
Entries in FASTA	82 492 (proteins)	3 255 122 (peptides)	75 410 898 (peptides)	82 492 (proteins)	3 255 122 (peptides)	52 244 303 (peptides)
Time for FASTA Generation	-	1h 15m 8s	20h 6m 19s	-	1h 14m 54s	11h 4m 32s
Mean (STD) time for Identification per Measurement	5m 47 s (51s)	4m 15s (36s)	1h 44m 4s (13m 40s)	5m (1m 2s)	4m 5s (48s)	1h 15m 13s (13m 15s)
Overall running time of workflow	15m 57s	1h 26m 9s	1d 46m 24s	10m 16s	1h 22m 51s	14h 9s

References

[1] Tim Schmidt, Lukas Kuhn, Bob Price, Johan De Kleer, and Rong Zhou. A depth-first approach to target-value search. In *Symposium on Combinatorial Search (SOCS-09)*. Citeseer, 2009.
